# Bioorthogonal Mussel-Inspired Elastin-like Nanocoatings for Indwelling Devices

**DOI:** 10.1021/acsami.5c10327

**Published:** 2025-09-01

**Authors:** Sergio Acosta, Viktoriya Chaskovska, Ikram El-Maachi, Jenny Englert, María Puertas-Bartolomé, Stefan Jockenhoevel, José Carlos Rodríguez-Cabello, César Rodriguez-Emmenegger, Alicia Fernández-Colino

**Affiliations:** † Department of Biohybrid & Medical Textiles (BioTex), AME − Institute of Applied Medical Engineering, Helmholtz Institute, 9165 RWTH Aachen University, 52074 Aachen, Germany; ‡ Bioforge Laboratory (Group for Advanced Materials and Nanobiotechnology), Laboratory for Disruptive Interdisciplinary Science (LaDIS), CIBER-BBN, Edificio LUCIA, 16782 Universidad de Valladolid, 47002 Valladolid, Spain; § DWI Leibniz Institute for Interactive Materials, Forckenbeckstraße 50, 52074 Aachen, Germany; ∥ Chair of Biotechnology, 9165 RWTH Aachen University, 52074 Aachen, Germany; ⊥ Institute for Bioengineering of Catalonia (IBEC), The Barcelona Institute of Science and Technology (BIST), Carrer de Baldiri Reixac 10-12, 08028 Barcelona, Spain; # Institució Catalana de Recerca i Estudis Avançats (ICREA), Passeig Lluís Companys 23, 08010 Barcelona, Spain; ∇ Biomedical Research Networking, Center in Bioengineering, Biomaterials and Nanomedicine, The Institute of Health Carlos III, 28029 Madrid, Spain

**Keywords:** elastin-like recombinamers, biofunctional coatings, surface functionalization, DOPA, click chemistry

## Abstract

Medical devices such as vascular grafts, stents, and catheters are crucial for patient treatment but often suffer suboptimal integration with host tissues due to the nature of their surfaces. The materials commonly used, including metals and synthetic polymers, frequently lead to undesired immune responses and device failure. In this context, coating their surfaces with designer proteins has arisen as a promising strategy to improve the device’s biointegration. Here, we present a bioinspired method for coating biomaterial surfaces with protein-engineered polymers designed to mimic tailored functions from the native extracellular matrix (ECM). Combining mussel-inspired catechol chemistry with bioorthogonal click chemistry, we developed a modular grafting method for the surface functionalization of metallic and polymeric implants using a bifunctional peptide containing azide and DOPA (3,4-dihydroxyphenylalanine) groups. This simple dip-coating process enabled the fabrication of bioactive elastin-like coatings with precise peptide presentation. The results reveal enhanced bioactivity and cytocompatibility, as evidenced by improved endothelial cell adhesion, proliferation, and heparin-binding capacity on coated surfaces. The versatility and effectiveness of this bioorthogonal coating method suggest significant potential for creating implant surfaces tailored to diverse clinical applications.

## Graphical abstract

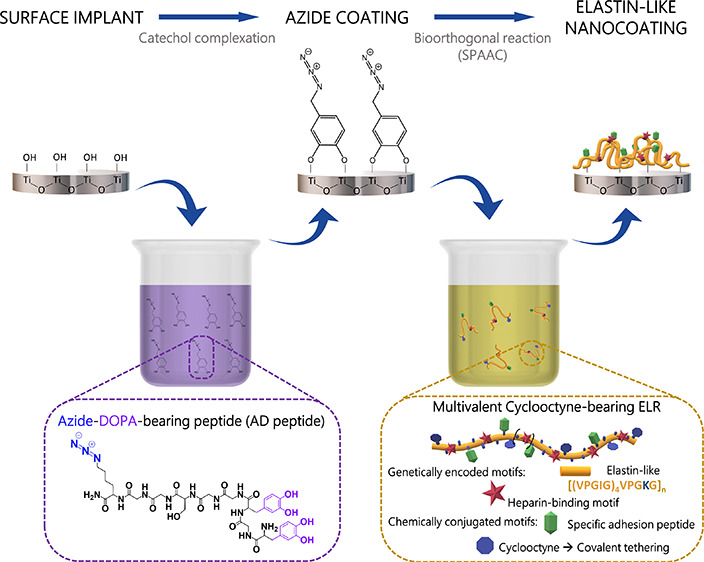

## Introduction

The biomaterials commonly used for the development of medical implants, such as metals, ceramics and synthetic polymers, are often nonbiodegradable and lack bioactivity.^1^ These materials are capable of effectively replacing the mechanical functions of damaged tissues, but they often fail to actively interact with the surrounding biological environment, including host cells and the immune system.^2^ Their implantation often results in inadequate integration with host tissues, thus triggering undesirable cellular responses, including fibrous encapsulation, chronic inflammation or thrombotic events among others. These reactions can ultimately lead to complications and implant failure. For instance, cardiovascular devices, such as metal stents or polymeric vascular grafts, frequently fail due to thrombotic events,^2^ while percutaneous implants are prone to complications arising from poor integration with soft tissues.^3^ All these issues arise from the inability of biomaterial surfaces to mimic the biological properties of the extracellular matrix (ECM) of the tissues they are intended to replace, thus impeding proper device integration.

To address these challenges, one of the most promising strategies focuses on developing bioactive coatings capable of camouflaging implant surfaces by mimicking key properties of the native ECM.^4,5^ Biomimetically coated devices aim to integrate the mechanical advantages and wear resistance of traditional biomaterials with the biological functionality required for their successful integration.^6,7^ These coatings are designed to interact with host cells, regulate immune responses, and create a local microenvironment that promotes implant integration and tissue healing.^3,8^

A common strategy to achieve bioactive surfaces is through the incorporation of ECM-derived peptides or proteins, which provide the biological cues necessary for cell adhesion and tissue repair. Biomimetic coatings that incorporate full ECM proteins, such as fibronectin or tropoelastin, have demonstrated the ability to drive stem cell proliferation, immune modulation, and tissue regeneration.^9–11^ However, maintaining the structural integrity and biological activity of these proteins on implant surfaces presents a major challenge. Conventional adsorption methods can lead to protein denaturation and decreased functionality.^12,13^ As a result, alternative strategies have emerged, such as tethering short bioactive peptides derived from ECM proteins.^3,14,15^ These peptides retain key biological functions while offering greater stability and ease of processing. For example, general cell-adhesive peptides, such as RGD (Arg-Gly-Asp), can promote cell migration and adhesion, while antimicrobial and immunomodulatory peptides can be exploited for infection prevention and inflammation control.^15,16^

Despite these advances, developing coatings that mimic the viscoelastic properties of the native ECM and simultaneously present multiple bioactive cues in a controlled, reproducible, and spatially organized manner remains a significant challenge. This is particularly important for applications where multiple biological processes must occur simultaneously, such as vascular implants requiring endothelialization while preventing thrombosis, or orthopedic implants requiring tissue integration while minimizing infection risk.^17,18^

Designer proteins are synthetic proteins that are specifically engineered with tailored viscoelasticity, bioactive sequences and reactive groups for functionalization. Elastin-like recombinamers (ELRs) represent a promising class of designer proteins for achieving such control, versatility and biomimicry. These recombinant polymers (“recombinamers”) are inspired by natural elastin, a key component of the ECM.^19^ ELRs are typically based on the repetition of the tropoelastin-derived peptide VPGXG, where X represents any amino acid except l-proline. ELRs mimic many of the unique biomechanical properties of elastin, such as elasticity, resilience, and excellent biocompatibility, making them highly suitable for tissue engineering applications.^20,21^ Moreover, these protein-engineered polymers are nonimmunogenic and nonthrombogenic.^22–24^ When coated on a surface, they can reduce nonspecific protein fouling and prevent platelet activation,^25,26^ thus providing a favorable environment that supports tissue regeneration and modulates host responses. More importantly, their recombinant nature benefits from precise genetic engineering, enabling the incorporation of specific bioactive motifs or reactive amino acid residues directly into their sequence. This provides an unprecedented level of control over surface content and bioactive ligand density. Such degree of precision and versatility is difficult to achieve with synthetic or natural polymers.^27^ This high level of control allows the design of ELR coatings that are not only elastin–mimetic but also engineered with specific functionalities and bioactivities, including stimuli-responsiveness, cell-adhesive, antifouling or antibiofilm properties.^26,28–35^

Despite these advantages, the methods traditionally used for ELR immobilization present significant limitations that hinder their scalability and long-term stability. ELR-based coatings have primarily relied on physisorption or covalent attachment to biomaterial surfaces. Physisorption, while simple and widely applicable, relies on weak, noncovalent forces, and can lead to potential desorption under mechanical stresses, fluid flows or competitive protein adsorption.^36^ On the other hand, conventional covalent immobilization strategies typically require complex multistep reaction protocols,^29,37^ which can be difficult to scale up for industrial applications. Moreover, many reported covalent strategies rely on reactive groups that are prone to hydrolysis or degradation, such as organosilanes, limiting their long-term stability in aqueous environments.^29,38^ Consequently, there is a clear need for alternative strategies that enable the anchoring of protein-engineered polymers to diverse biomaterial surfaces, while being facile and scalable.

In this sense, incorporating catechol residues to bioactive peptides represents a facile and minimalistic approach for the fabrication of robust and functional coatings on implant surfaces.^39,15^ Recent studies have exploited the combination of mussel-inspired catechol residues with azide or cyclooctyne groups to enable biorthogonal “click” immobilization of biological effectors, such as small peptides or extracellular vesicles.^40,41^ These approaches have demonstrated improvements in implant integration and modulation of immune response. Specifically, nitric oxide-generating organoselenium and an endothelial progenitor cell-targeting peptide were clicked onto a stent to promote reendothelialization and inhibit thrombosis and smooth muscle cell proliferation, directly addressing complications like in-stent restenosis. Similarly, extracellular vesicles have been immobilized on titanium substrates to enhance osseointegration and immune modulation. However, the applicability of these approaches to large protein polymers, with the concomitant advantages of broader structural and functional versatility, remains elusive.

In this study, we implement a bifunctional grafting peptide that combines catechol-adhesion chemistry with biorthogonal ‘click’ chemistry to enable for the first time ELR-anchoring into implant surfaces, in a facile, scalable and stable manner. Inspired by the adhesive properties of mussel foot proteins, we designed a grafting peptide bearing azide and catechol groups. Catechol chemistry enables robust attachment to a wide range of biomaterial surfaces,^42^ including metals, ceramics and polymers,^43–45^ while the click chemistry allows precise and catalyst-free tethering of the ELRs on the surface. This modular approach ensures stable, uniform, and versatile coating deposition with precise control over bioactive peptide presentation.

## Experimental Section

### ELRs and Peptides Production

The ELRs were produced recombinantly and modified chemically for click chemistry. First, the encoding gene for the nHB ELR was purchased from NZYTech (Portugal) and inserted in a pET-25b (+) vector for expression in the *E. coli* BLR (DE3) strain. Bacterial fermentation was carried out in an autoinduced TB medium in a 15-L fermentor (Applikon biotechnology, USA) overnight. Afterward, the bacteria were collected and pelleted at 4 °C in saline buffer (20 mM Tris, 200 mM NaCl, pH 7.5) and disrupted in Tris-EDTA buffer. The ELRs were then purified by inverse transition cycling (ITC) using 1.5 M NaCl for precipitation at 40 °C and ultrapure water for resolubilizing the pellets overnight at 4 °C. After four purification cycles, the purity was verified by SDS-PAGE, and the ELR solutions were dialyzed against 25 L deionized water (three changes) and 25 L ultrapure water (one change).

For the fabrication of the coatings via the biorthogonal approach, the ELRs were modified with cyclooctyne groups as previously reported.^46^ To that end, the ELRs were dissolved in anhydrous dimethylformamide (DMF) at a final concentration of 20 mg mL^–1^ under an N_2_ atmosphere, and incubated with (1R,8S,9S)-bicyclo[6.1.0]­non-4-yn-9-ylmethyl succinimidyl carbonate (GalChimia) for 60 h at RT to modify eight Lys residues per chain. The resulting product was then precipitated with diethyl ether and washed three times with acetone. The product was dialyzed (three times against distilled water and once against ultrapure water), filtered for sterilization (0.22 μm Nalgene, Thermo Fisher Scientific, USA), lyophilized, and stored at −20 °C for long-term use.

To explore the potential of combining this immobilization approach with layer-by-layer (LbL) assembly, azide functional groups were introduced into the nHB ELR backbone by modifying primary amines of lysine residues with 2-azidoethyl (2,5-dioxopyrrolidin-1-yl) carbonate (GalChimia). The reaction was carried out following the same protocol and stoichiometry used for cyclooctyne modification.The peptides AD [(DOPA)-G-(DOPA)-GGSGGK­(N_3_)-NH_2_] and TPS [TPSLEQRTVYAKGGGGK­(N_3_)-NH_2_] were purchased from CASLO (Denmark) with purities of 92.92% and 99.59%, respectively.

The HB-TPS polypeptide was obtained by conjugating the TPS peptide to the HB backbone by click chemistry. To that end, HB was dissolved in an ethanol solution in DI water (50% v/v) at 20 mg mL^–1^, and incubated with three equivalents of the TPS peptide at RT overnight. The product was then dialyzed against ultrapure water (four changes), filtered and lyophilized for long-term storage. The number of peptides introduced was estimated by MALDI-ToF (Figure S1 and Table S2, Supporting Information).

### Fabrication and Characterization of the Coatings

Protein-engineered coatings were fabricated on model TiO_2_ surfaces. Commercially pure titanium (Ti) grade II disks (10 mm diameter) were purchased from Baoji Dynamic Trading (China), grounded and polished to a mirror-shine using an ATM Saphir550 polishing machine (Institute of Mineral Engineering, RWTH Aachen). To create a robust TiO_2_ coating, the Ti disks were etched (5 M NaOH, 60 °C, 16 h) and cleaned with DI water, isopropanol, and acetone, as previously described.^29^

TiO_2_ surfaces were functionalized by dip-coating. First, the disks were incubated in a grafted (100 μΜ solution of AD peptide solution in DI water overnight at room temperature. Prior to incubating with the ELR solution, the disks were ultrasound cleaned three times in DI water and then incubated in 200 μΜ solution of ELR in 50% ethanol overnight at room temperature. The disks were soaked in a sonication bath three times in 50% ethanol to remove physisorbed molecules.

The atomic composition of the nanocoatings was determined by X-ray photoelectron spectroscopy (XPS) in a PHI 5701 spectrometer equipped with a multichannel hemispherical analyzer (SCAI, University of Malaga, Spain). Measurements were taken with an Mg excitation source (300 W, 15 kV, 1253.6 eV, beam diameter 720 μm). The spectrometer’s energy scale was calibrated using Cu 2p3/2, Ag 3d5/2 and Au 4f7/2 photoelectron lines at 932.7, 368.3, and 84.0 eV, respectively. The binding energy of photoelectron peaks was referenced to C 1s core level for adventitious carbon at 284.8 eV. High-resolution spectra were recorded at a given takeoff angle of 45 by a concentric hemispherical analyzer operating in the constant pass energy mode at 29.35 eV.

The wettability of the surfaces was measured by water contact angle (WCA) using the sessile-drop method. Ultrapure water droplets (vol 3 μL) were deposited on the surfaces and measured and analyzed with an automatic contact angle and contour analysis system (OCA 20, DataPhysics Instruments GmbH, Germany).

Related to the coating of the polymeric surfaces, medical-grade thermoplastic polyurethane (Durathane ALC) was kindly provided by ICP-DAS Biomedical Polymers (Taiwan), and polyethylene terephthalate (PET) sheets were purchased from Mytech (Bulgaria). Samples of 10 × 10 mm were treated with argon (Ar) plasma for 10 min to create reactive functional groups on the surface and functionalized with the ELRs using the bifunctional azide-DOPA peptide by dip coating following the same two-step procedure used for metallic substrates. After the dip-coating procedure, the samples were thoroughly rinsed in a sonication bath with ultrapure water three times to remove unbound material. The functionalized surfaces were then vacuum-dried and analyzed by XPS to confirm successful ELR tethering and determine the elemental composition.

### Morphological Characterization

Peptide and ELR coatings were prepared on 6 mm Ti discs for surface topography analysis using an MFP-3D-BIO AFM (LTI, University of Valladolid) equipped with AC160 cantilevers and operated in tapping mode. Representative 2 × 2 μm areas were imaged for each sample. Surface roughness values were calculated using Gwyddion software. Domain diameters were quantified using ImageJ, with between 110 and 140 individual domains measured per surface type.

### Real-Time Monitoring of the Coating Fabrication and Heparin Adsorption

Quartz crystal microbalance with dissipation monitoring (QCM-D) and surface plasmon resonance (SPR) were used to monitor in situ the formation of the protein coating as well as for the characterization of the heparin adsorption.

QCM-D (Q-Sense, Biolin Scientific AB, Sweden) was used for monitoring the frequency and dissipation changes on Qsensors coated with titanium (Ti; QSX 310, Biolin Scientific). The sensors were cleaned following the manufacturer instructions. All solutions were run at a flow rate of 20 μL min^–1^. Frequency and dissipation data were acquired with QSoft401 (version 2.7.3.883, Biolin Scientific, Sweden) and analyzed using Qsense Dfind software (version 1.2.1, Biolin Scientific, Sweden). For mass absorption estimation, frequency and dissipation shifts at harmonics 5, 7, and 9. Depending on the characteristics of each layer, the Sauerbrey model or the viscoelastic Voigt model was applied using QSense Dfind.

SPR experiments were carried out on the MP-SPR Navi 210A VASA (BioNavis, Tampere, Finland) with a dual-channel microfluidic flow cell. The system was operating using the SPR-Navi Control software (Version 4.2.5.2), and the acquired data, recorded at a wavelength of 670 nm, were analyzed with the SPR-Navi Data Viewer (Version 4.3.5.2). The adsorbed protein mass was calculated based on the changes in refractive index (ΔμRIU).

To monitor the in situ anchoring of the ELRs using the bioorthogonal approach, the following sequence of events was followed: (i) The sensor was stabilized in 50% ethanol until a stable baseline was defined; (ii) AD peptide solution at 100 μM in 50% ethanol was run; (iii) a 50% ethanol rinsing step was performed; (iii) ELR solution at 200 μM was run; (iv) final rinsing step with 50% ethanol.

To assess the feasibility of integrating this coating approach with LbL fabrication, an alternating deposition sequence of azide- and cyclooctyne-functionalized ELRs (HB) was performed using QCM-D. The process involved the following sequence: (i) stabilization of the sensor with a constant flow of 50% ethanol solution until a stable baseline is reached, (ii) AD peptide solution, (iii) rinsing with 50% ethanol, (iv) bioorthogonal tethering of the cyclooctyne-bearing ELR, (v) rinsing with 50% ethanol, (vi) tethering of the azide-bearing, (vii) rinsing with 50% ethanol, (viii) bioorthogonal tethering of the cyclooctyne-bearing ELR, and (ix) final rinsing with 50% ethanol.

To quantify the capacity of the ELR coatings to adsorb heparin, the sequence of events was as follows: (i) Stabilization of the sensor with PBS; (ii) heparin sodium salt (Carl Roth) solution at 1.5 mg mL^–1^ in PBS was run until reaching the maximum frequency and dissipation changes; (iii) a rinsing step with PBS.

### Stability of the Coating

To assess the stability of the ELR coatings, the HB ELR was fluorescently labeled with FITC-NHS (Thermo Fisher). Briefly, cyclooctyne-functionalized HB was dissolved in anhydrous DMF at a concentration of 10 mg mL^-1^. Under a nitrogen atmosphere, a stoichiometric amount of FITC-NHS was added to achieve the conjugation of approximately one fluorophore per ELR chain. The reaction was performed at room temperature under constant stirring for 24 h. The labeled polymer was subsequently dialyzed against distilled water (4 changes) and Milli-Q water (1 change) using dialysis tubing with a molecular weight cutoff of 12,000 Da. The product was then lyophilized.

Fluorescent coatings were prepared on 6 mm Ti discs using the same two-step surface functionalization protocol described above, substituting HB with FITC-labeled HB (FITC-HB). To evaluate coating stability under physiological conditions, the samples were subjected to mechanical challenge via ultrasonication in PBS for 5 min (Selecta Ultrasonic bath, model 3000683, 110W), followed by incubation in PBS at 37 °C. Discs were collected at defined time points (0, 3, and 7 days) and analyzed using confocal laser scanning microscopy (Leica SP8).

### Cell Isolation and Expansion

The biological response of the ELR coatings was evaluated with primary vascular cells. hUVECs were isolated from human umbilical cords as previously described.^47^ Human umbilical cords were obtained after written informed consent at University Hospital Aachen, Germany. They were provided by the RWTH Aachen University Centralized Biomaterial Bank (cBMB) according to its regulation and following RWTH Aachen University Medical Faculty Ethics Committee approval (cBMB project number 323). hUVECs were cultured in endothelial growth medium 2 (EGM2; PromoCell, Germany) supplemented with 1% FCS, epidermal growth factor, basic fibroblast growth factor, insulin-like growth factor, vascular endothelial growth factor 165, ascorbic acid, heparin, and hydrocortisone. The cells were expanded under standard culture conditions in a humidified 5% CO_2_ atmosphere at 37 °C. Both cell types were used in passages 2–4 for all the experiments.

hCB-EPCs (passage 3, CELLvo, StemBioSys) were cultured according to the manufacturer’s instructions in DMEM medium supplemented with 20% FCS, 1% l-glutamine, 10 ng mL^–1^ basic fibroblast growth factor, and 20 ng mL^–1^ epithelial growth factor.

### In Vitro Biological Response

To evaluate the effects of ELR coatings on cell adhesion and proliferation, TiO_2_ disks, pristine or functionalized with nonbioactive ELR (IK), and bioactive ELR coatings (HB and HB-TPS) were used.

Adhesion assays: Samples were pretreated with 1% (w/v) BSA in PBS for 1 h at room temperature to promote specific adhesion. A cell suspension of 7 × 10^4^ cells mL^–1^ was prepared, and a cell density of 2.5 × 10^4^ cells cm^2^ were seeded onto the disks. Samples were then incubated for 2 h at 37 °C under standard culture conditions. After incubation, nonadherent cells were removed by washing with PBS, and adherent cells were fixed with 4% paraformaldehyde (PFA) for 20 min.

For immunostaining, samples were permeabilized with 5% normal goat serum and 0.1% Triton X-100 in PBS. Endothelial cell marker (CD31) was labeled by immunostaining. Samples were incubated with the primary antibody solution (CD31 mouse antihuman, Thermo Fisher, 1:100 dilution in PBS supplemented with 1% BSA, 0.1% sodium azide) 45 min at room temperature followed by an incubation at 4 °C overnight. Excess primary antibody was removed by sequential washing with PBS for 2, 5, 15, and 30 min. Secondary antibody staining was performed using Alexa Fluor 568 goat antimouse IgG (Thermo Fisher, 1:400 dilution) for 1 h at room temperature, followed by the same washing protocol as for the primary antibody. F-actin filaments were stained with Phalloidin-A488, and nuclei with DAPI. Images were acquired using a fluorescence microscope (AxioObserver Z1; Carl Zeiss GmbH) equipped with epi-illumination. Image analysis was conducted with ImageJ software.

Proliferation assays: Cell proliferation was quantified using the Cell Counting Kit-8 (CCK-8) assay kit. Cells were seeded on the coatings in complete culture medium without BSA treatment, allowing for early cell adhesion on all surfaces (Figure S7, Supporting Information) and incubated for 24 and 72 h under standard culture conditions. The samples were then incubated for 3 h in CCK8 solution and the absorbance at 450 nm was recorded using a microplate reader (Infinite M200, Tecan) to determine the number of viable cells.

### Statistical Analysis

All the reported experiments were performed at least in triplicates. Statistical analysis were performed with GraphPad Prism software (version 8.4.3). One-way analysis of variance (ANOVA) using the Tukey’s multiple comparison test was used to analyze statistical differences. A *p*-value of less than 0.05 was considered to be statistically significant (*p < 0.05, **p < 0.01, ***p < 0.001). Any value of *p* > 0.05 was defined as nonsignificant (ns).

## Results and Discussion

### Design and Physicochemical Characterization of Protein-Engineered Coating

We successfully engineered and recombinantly produced an ECM-mimetic protein polymer based on the pentapeptide repetition (VPGXG)_120_, where X = Ile/Lys in a 4:1 ratio (nHB ELR). The incorporation of hydrophobic isoleucine residues contributes to lowering the phase transition temperature below physiological levels,^48^ while lysine residues, with their primary amine groups on the side chain, allow for chemical derivatization with NHS-esters. In this case, 9 out of the 24 lysines per chain were modified with cyclooctyne groups to enable anchoring on azide-modified surfaces via strain-promoted azide–alkyne cycloaddition (SPAAC) (Figure 1a, Table S1 and Figure S1, Supporting Information). The resulting protein-engineered polymer, named heparin-binding (HB) ELR, also incorporates the bioactive peptide PRRARV in its sequence. PRRARV peptide is part of the C-terminal heparin-binding domain of fibronectin, involved in the interaction with membrane proteoglycans and heparin.^49^

**Figure 1:**
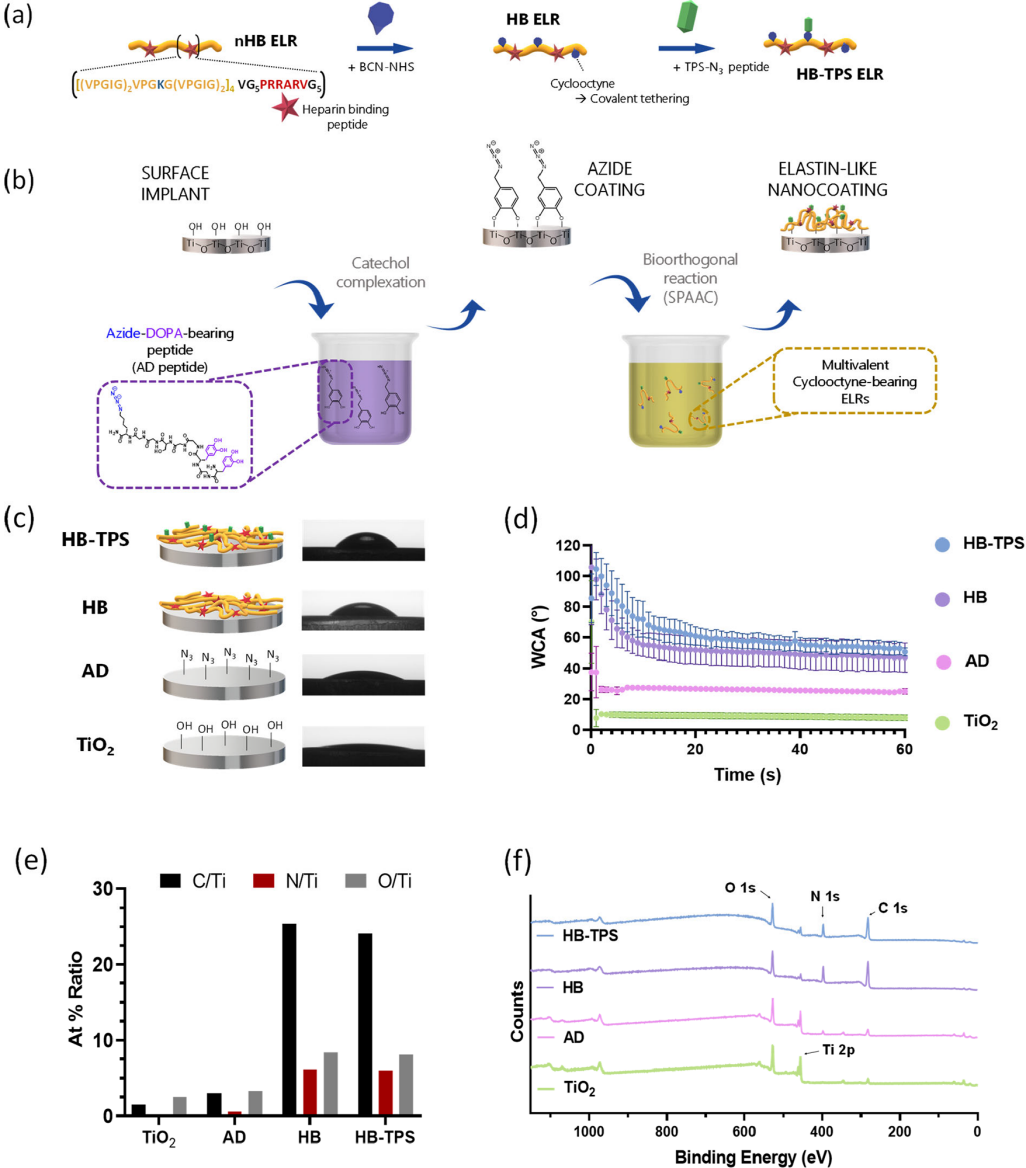
(a) Schematic representation of the chemical derivatization of the nonmodified heparin-binding (nHB) ELR. Cyclooctyne groups were conjugated to the lysine side chains, yielding the clickable HB ELR, which was further functionalized with the cell-adhesive TPS peptide via bioorthogonal click chemistry to obtain HB-TPS ELR. (b) Illustration of the surface coating strategy and (b–e) physicochemical characterization of the surfaces after different modification steps. By simple immersion, azide-bearing peptides are grafted on model TiO_2_ surfaces via a mussel-inspired catechol complexation reaction, thus creating anchoring sites for tailor-made protein polymers. In a subsequent immersion step, ELRs are covalently tethered through bioorthogonal catalyst-free reaction (strain-promoted alkyne–azide cycloaddition, SPAAC). (c) Scheme of the resulting nanocoatings and representative images of water drops on the surfaces after 30 s. (d) Water contact angle (WCA) measurements of the surfaces after each modification step (*n* = 15). (e) X-ray photoelectron spectroscopy (XPS) semiquantitative analysis showing relative atomic ratios of key elements (C, N, and O) with respect to the substrate’s primary element (Ti) (*n* = 3). (f) Representative XPS survey spectra of the modified surfaces. Peaks for elements of interest are marked.

Additionally, HB ELR was further functionalized with the TPS peptide, which was anchored to their cyclooctyne groups by click chemistry (Figure 1a, Tables S1 and S2, Supporting Information). TPS peptide has been identified as a selective adhesion peptide for endothelial progenitor cells (EPCs),^50^ and has proven efficacy in promoting the homing of circulating EPCs while preventing platelet adhesion on cardiovascular implants.^51^ Its incorporation into the ELR coating not only provides EPC-selective functionality but also demonstrates the versatility of our modular platform. By combining genetically encoded bioactive and structural motifs with chemical functionalization via click chemistry, it allows for precise and customizable control over cell adhesion and host response modulation on implant surfaces. To ensure strong and stable adhesion of ELRs to biomaterial surfaces, we devised a functionalization strategy (Figure 1b) that combines the universal adhesive properties of the mussel-derived amino acid DOPA^52^ with the orthogonality of click chemistry. To that end, we designed a bifunctional anchoring peptide (azide-DOPA peptide, or AD peptide, Table S2, Supporting Information). This peptide incorporates two DOPA residues at one end and an azide group at the other, connected by a flexible linker of five amino acids (i.e., GGSGG). The presence of two DOPA residues has been shown to be sufficient for peptide tethering on biomaterial surfaces,^44,53^ while the flexible linker ensures optimal exposure of the azide group, once the peptide is anchored to the substrate.^54^ In the initial immersion step into the AD peptide solution, the surface of the implant is modified with azide groups via the catechol-mediated adhesion, providing anchoring sites for the biorthogonal click reaction with cyclooctyne-bearing ELRs introduced in a subsequent immersion step.

The physicochemical characterization of the surfaces after each step in the coating fabrication process revealed the effective tethering of the AD peptide and the ELRs to the surface (Figure 1c–f).

Wettability of the modified surfaces was assessed by water contact angle (WCA) analysis, which showed significant differences. Pristine TiO_2_ surfaces were initially superhydrophilic (WCA < 10°) and maintained low values throughout the test, with a WCA of 9.0 ± 1.9° after 30 s (Figure 1c). After the first immersion step on AD solution, the hydrophilicity of the surface slightly decreased (WCA = 26.3 ± 1.1°), confirming AD peptide’s anchoring. Subsequent immersion on the ELRs’ solution further increased the hydrophobicity of the surfaces, speaking for the anchoring of the ELRs to the AD peptide on the materials surface. The wettability profiles for both recombinamers showed that HB coating was slightly more hydrophilic (WCA = 50.1 ± 8.6°) than HB-TPS coating (WCA = 57.3 ± 4.8°) (Figure 1c).

Moreover, the evolution of the WCA measurements over time varied significantly between the surfaces (Figure 1d). While the pristine TiO_2_ and AD-coated surfaces exhibited quasi-static WCA values throughout the measurement time (60 s), substantial changes were observed for the ELR coatings. Initially, upon deposition of the water drop, the coatings appeared hydrophobic (WCA > 90°), likely due to the high content of hydrophobic amino acids in their sequence (Table S1, Supporting Information). As the measurement progressed, the surface became increasingly hydrophilic. This behavior can be attributed to the flexibility of the ELR chains,^29^ which possibly allowed for the rearrangement of amino acid side chains within the coating, exposing more hydrophilic side groups and thereby rendering the surface hydrophilic. This observation highlights the dynamic response in aqueous solution of ELR coatings.

In parallel, the surface topography of the coatings was evaluated by AFM (Figure S2, Supporting Information). The pristine TiO_2_ showed a nanostructured surface with defined peaks as a consequence of the chemical etching.^55^ Upon surface modification (with AD peptide, HB ELR, and HB-TPS ELR), the topography seemed to became slightly more homogeneous and rounded (Figure S2a). Quantitative analysis of the AFM images showed that surface roughness did not differ significantly between the nanocoated samples and the pristine TiO_2_ surface. While the presence of a coating tended to increase roughness, this difference was not statistically significant (Figure S2b,c). However, significant differences were observed in the lateral dimensions of surface features. While pristine TiO_2_ showed narrow and sharply defined domains, the average grain diameter increased after coating the surfaces with AD, HB, and HB-TPS. Image analysis revealed changes in the mean domain size from approximately 52 nm on TiO_2_ to 93–105 nm on the modified surfaces (Figure S2d), indicating lateral spreading of the topographic features upon peptide and ELR deposition. Further surface analysis by XPS revealed a progressive decrease in the Ti 2p signal upon AD peptide or ELRs solution immersion, accompanied by an increase in N 1s and C 1s (Figure 1e,f). This results in an elevated N/Ti and C/Ti atomic ratio, confirming the successful immobilization of bioactive ELRs on the metal surface.

High-resolution XPS spectra revealed the progressive evolution of the peaks after each functionalization step, which confirmed the successful coating of the surface with AD peptides and ELRs (Figure S3, Supporting Information). In the C 1s region, the pristine TiO_2_ surface showed a single peak centered at ∼284.6 eV, attributed to environmental aliphatic carbon contamination. Upon peptide and ELR deposition, this peak broadened probably due to additional contributions from C–N and C–OH groups present in the peptide and protein backbone. Moreover, a secondary peak also appeared at ∼288.0 eV, indicative of carbonyl groups (C=O) from the peptide bond. The O 1s region also showed a shift from a dominant lattice oxygen peak (∼530.0 eV) in pristine TiO_2_ to the appearance of a shoulder at ∼531.5 eV after AD tethering, attributed to amide carbonyls and phenolic O. This signal increased further following ELRs immobilization, supporting the successful formation of the protein coating. Additionally, differences were found in the N 1s region. Whereas no detectable signal was observed on TiO_2_, a clear peak at ∼399.5 eV appeared after AD peptide deposition, assigned to amide nitrogen. A slight broadening toward a higher binding energy (∼401 eV) might indicate minor contributions from azide functionalities present in the AD coatings. After ELR immobilization, the N 1s peak increased significantly in intensity, confirming a higher nitrogen content. Finally, the progressive attenuation of Ti 2p_3/2_ and Ti 2p_1/2_ peaks at ∼458.5 and ∼464.2 eV, respectively, through the functionalization steps further supports the gradual coverage of the TiO_2_ surface by an ELR layer. The ELR-coatings also demonstrated stability under mechanical and physiological challenges (Figure S4), thus suggesting that the combination of catechol-based adhesion with click chemistry provides superior hydrolytic stability for ELR immobilization compared to conventional organosilane-based approaches.^29^ This result is consistent with previous findings that highlight the robustness and durability of this dual-chemistry strategy for surface functionalization.^39,56^

To further validate the versatility of this coating strategy, we applied it to synthetic polymeric materials commonly used in permanent implants, such as cardiovascular devices and ligament replacements. We selected thermoplastic polyurethane (TPU), and polyethylene terephthalate (PET) as model nonbioactive synthetic polymers.^57,58^

The XPS results revealed a progressive increase in the N 1s signal following the application of AD and ELR solutions (Table 1 and Supporting Information, Figure S5). This characteristic peak, typically associated with proteins and notably absent in the pristine polymeric substrates, provides strong evidence for the successful deposition of the ELR on the surface. The nitrogen content reached approximately 12 and 16% for TPU and PET, respectively, demonstrating the effectiveness of this approach for functionalizing polymeric surfaces. These results highlight the adaptability of the azide-DOPA functionalization strategy, across different polymeric surfaces typically present in permanent implants.^42^

**Table 1:** Surface Composition (%) Determined by XPS after Each Modification Step (*n = 3*)

	C 1s	N 1s	O 1s
PET	70.93 ± 1.83	0.47 ± 0.16	28.77 ± 0.30
PET-AD	70.14 ± 2.73	10.32 ± 0.24	19.55 ± 2.98
PET-ELR	67.46 ± 0.09	15.71 ± 0.65	16.81 ± 0.55
TPU	82.25 ± 2.22	0.57 ± 0.67	17.18 ± 0.95
TPU-AD	71.83 ± 0.50	2.59 ± 0.13	25.59 ± 0.62
TPU-ELR	70.26 ± 1.26	12.22 ± 0.07	17.52 ± 1.19

### Real-Time Monitoring of the ELR Nanocoatings Formation

The coating fabrication process was in situ monitored using surface plasmon resonance (SPR) and quartz-crystal microbalance with dissipation (QCM-D) (Figure 2). SPR analysis revealed clear shifts in the refractive index upon each coating step (Figure 2bi,bii), indicating the attachment of both the AD peptide and ELRs onto the TiO_2_ sensor. This behavior was corroborated by QCM-D analysis (Figure 2ci,cii). QCM-D results showed a significant decrease in frequency after the infusion of the AD peptide (Δ*f*_7_ = 8.36 ± 2.81 Hz), which remained almost unchanged during the rinsing stage, indicating strong adsorption of the AD peptide onto the TiO_2_ substrate (Figure 2c). When the ELR solutions were infused, further decreases in frequency were observed for both ELRs. After the second rinsing step, the frequency stabilized at Δ*f*_7_ = 22.64 ± 6.40 Hz for HB coating and Δ*f*_7_ = 14.54 ± 7.93 Hz for HB-TPS coating, indicating a successful ELR anchoring to the surface. However, the dissipation values (Δ*D*), which reflect the viscoelastic properties of the adsorbed layer, followed a different trend. Although dissipation initially increased upon ELR infusion, it decreased during rinsing, ultimately reaching values of Δ*D* = 0.53 ± 0.47 × 10^–6^ for HB and Δ*D* = 0.48 ± 0.25 × 10^–6^ for HB-TPS coatings, respectively.

**Figure 2:**
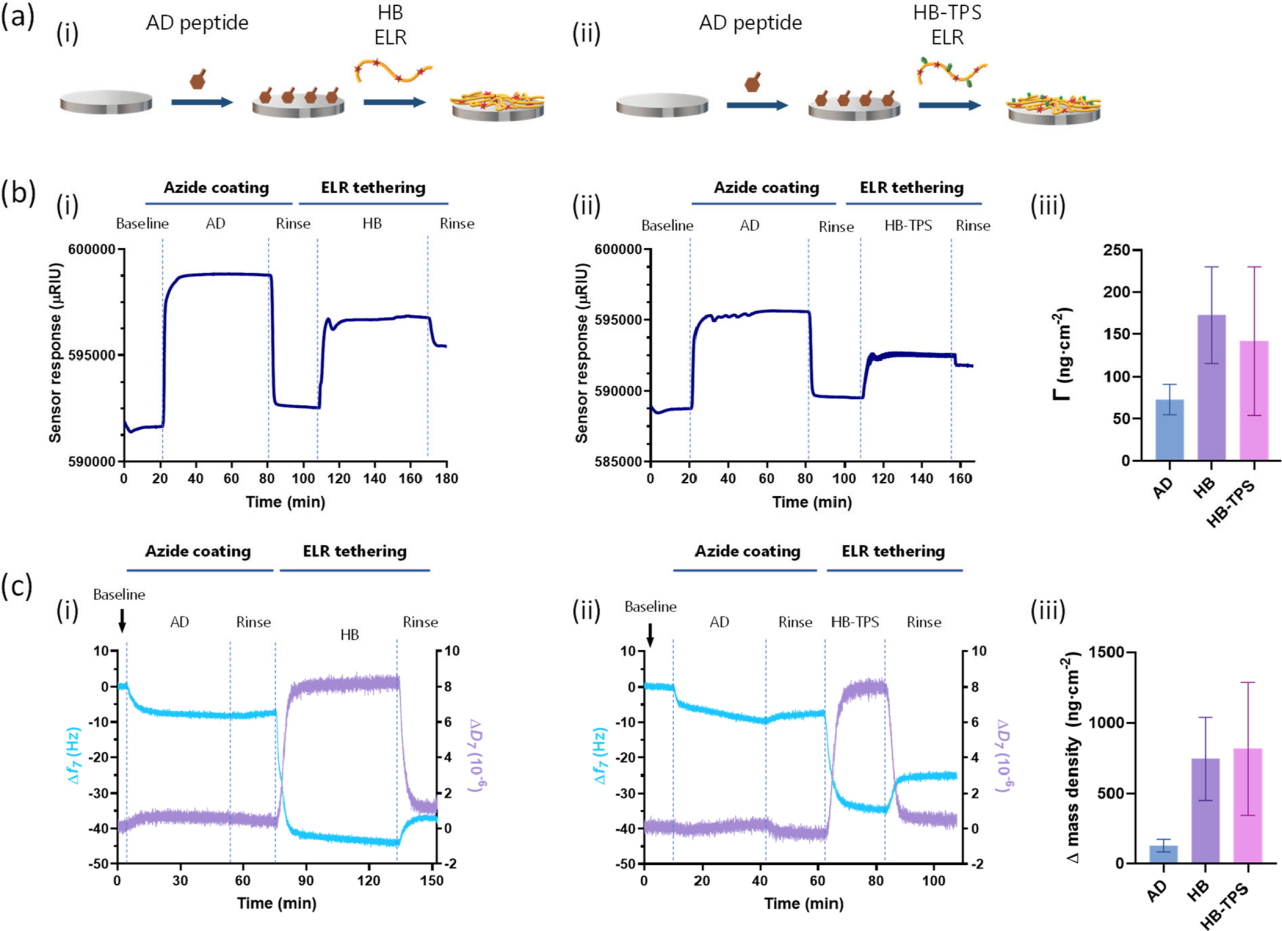
Real-time monitoring of the ELR nanocoatings formation. (a) Scheme of the immobilization process of (i) HB and (ii) HB-TPS ELRs on metal surfaces. Representative isotherm curves monitored by (b) surface plasmon resonance (SPR) and (c) quartz-crystal microbalance with dissipation (QCM-D). Graphs on the right show the average mass density deposited on the sensor estimated by SPR (b, iii) and QCM-D (c, iii), with error bars representing standard deviations (*n* = 3). The measurement cycle was, in both techniques, as follows: (1) baseline recorded in solvent solution (50% ethanol in ultrapure water); (2) addition of the AD peptide to create an azide coating; (3) rinsing with 50% ethanol; (4) bioorthogonal tethering of the ELR on the surface; (5) final rinse with 50% ethanol.

The ELR mass density anchored to the surface was then estimated (Figure 2biii,ciii), with values of approximately 750 ng cm^–2^ from QCM-D and around 150 ng cm^–2^ from SPR measurements. These differences in mass density arise from the fact that QCM-D is sensitive to the tethered ELRs and the coupled solvent molecules, leading to a higher mass uptake compared to the optical mass detected by SPR.^59^

The Δ*D* values observed in QCM-D are lower than those typically reported when ELRs are either physisorbed^60^ or covalently tethered via single-point attachment in an oriented way.^34,61^ The observed difference suggests that in our system, ELRs are immobilized through multipoint surface attachment enabled by the high density of azide groups on the surface. Bonding performance analysis (Table S3, Supporting Information) revealed a consistently higher density of azide groups compared to cyclooctyne moieties on the ELRs. This stoichiometric excess of azide functionalities likely favors multiple cycloaddition events per ELR molecule.

Such multipoint attachment is expected to result in a more compact and less dissipative coating compared to conventional physisorption or single-point bonding methods. The constrained molecular conformation imparted by multiple anchoring sites enhances the rigidity and mechanical robustness of the ELR layer, an advantage for applications requiring durable, low-dissipation biomaterial surfaces.

These differences in the density of reactive groups available for click reactions, along with the reduced variations in Δ*D* values observed in QCM-D compared with previous studies, prompted us to further investigate the involvement of cyclooctyne groups in the covalent attachment of the ELR to the surface. Specifically, we aimed to determine whether all cyclooctyne groups were consumed during this process or if some remained available for further functionalization with other bioactive molecules or for integration with additional fabrication methods, such as layer-by-layer (LbL) assembly.^32,62^ To investigate this, we explored the feasibility of fabricating multilayer LbL click-coatings based on ELRs anchored to the implant surface via the AD peptide.

QCM-D analysis (Figure S6, Supporting Information) showed a progressive decrease in frequency (Δ*f*_7_) after each coating step reaching values of Δ*f*_7_ ∼ −170 Hz after the last rinsing step, indicating the progressive protein mass deposition and therefore a sequential ELR layer formation. Additionally, dissipation values (Δ*D*_7_) increased progressively with each layer, reaching approximately 30 × 10^–6^ after three ELR layers, which indicates the formation of a viscoelastic coating.^32^ Therefore, these results demonstrate that not all cyclooctyne groups are involved in anchoring the ELR to the surface, leaving groups available for covalent linkage through cycloaddition with other macromolecules functionalized with azide groups. Additionally, this fact suggests the versatility of this approach, and its combination potential to fabricate multilayered, stimuli-responsive films that mimic the ECM on implant surfaces.

### In Situ Heparin Adsorption on the Elastin-like Nanocoatings

Heparin adsorption on the ELR-based coatings was evaluated using QCM-D and SPR (Figure 3). In vitro results indicated that the ELR coatings can bind heparin from circulating fluid and retain it after rinsing (Figure 3). Although the frequency shifts observed by QCM-D were relatively small, the consistent retention of heparin postrinsing and the low dissipation values suggest the formation of a stable adsorbed layer (Figure 3b). This was corroborated by SPR analysis, with estimated absorbed heparin concentrations of approximately 40 and 30 ng cm^–2^, as measured by QCM-D and SPR respectively (Figure 3b,c).

**Figure 3:**
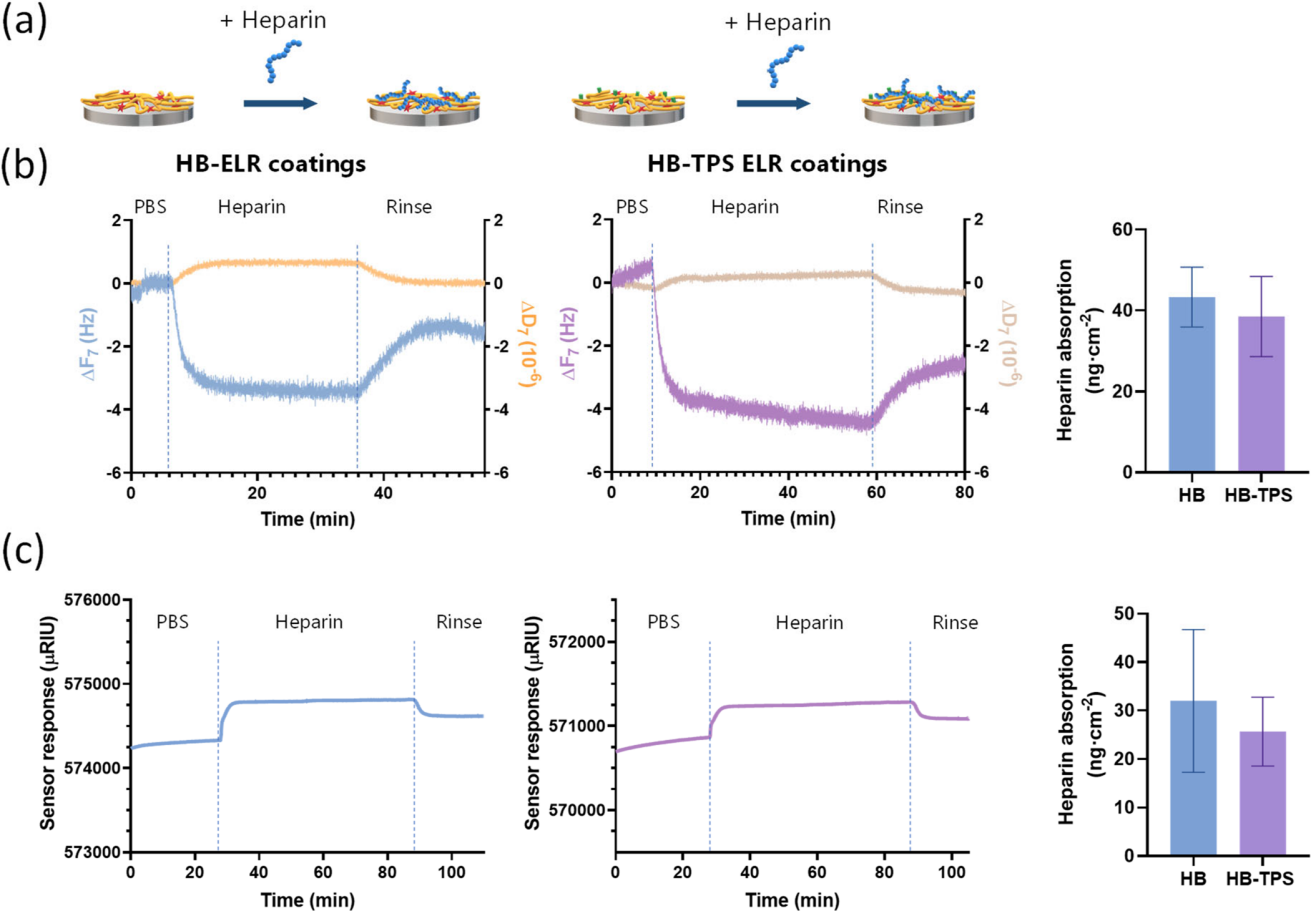
In situ heparin adsorption on the elastin-like nanocoatings onto TiO_2_ surfaces. (a) Schematic representation of the surface functionalization and subsequent heparin adsorption onto the ELR nanocoatings (HB and HB-TPS). (b) QCM-D measurements showing the changes in frequency (Δ*f*_7_) and dissipation (Δ*D*_7_) during heparin infusion and rinsing. (c) SPR measurements showing in refractive index upon heparin absorption. All measurements (*n* = 3) confirmed the successful immobilization of heparin on the ELR nanocoating.

The heparin adsorption was slightly lower in the HB-TPS coating, possibly due to steric or electrostatic hindrance provoked by the presence of the TPS peptide. However, this difference was not statistically significant.

The successful adsorption of heparin aligns with the presence of the PRRARV peptide within the ELR sequence, which is involved in the direct interaction with membrane proteoglycans and glycosaminoglycans, such as heparin.^49^ Importantly, this glycosaminoglycan-binding capability is biologically relevant, as it reflects the potential of ELR coatings to interact with endogenous heparin-like glycosaminoglycans in vivo. Such interactions may promote the binding of heparin-binding proteins, including growth factors, further enhancing the bioactivity of the ELR coatings. Moreover, the ability of the ELR coatings to act as a heparin reservoir suggests a potential role in the modulation of cellular responses, mimicking the behavior of fibronectin in the ECM.^54^Moreover, the recombinant nature of the ELR also enables to increase the density of the PRRARV motifs, paving the way to increase the amount of heparin to be bind to the coating, if necessary, according to the intended application. Notably, the modular design of ELR coatings allows for the incorporation of multiple bioactivities, either through genetic engineering or chemical derivatization, thereby expanding their applicability for tailoring the surface properties of medical implants to specific clinical needs.

### Cellular Response of the ELR Nanocoatings

The in vitro ability of the coatings to promote endothelialization was evaluated by measuring the adhesion (Figure 4a) and proliferation (Figure 4b) of two primary endothelial cell types: human umbilical cord endothelial cells (hUVECs) and human cord blood-endothelial progenitor cells (hCB-EPCs). Bioactive coatings presenting the fibronectin-derived peptide PRRARV (HB) or a combination of PRRARV and TPS (HB-TPS), were evaluated and compared with a nonbioactive ELR coating based on an ELR lacking cell-adhesive sequences (IK), previously reported.^63^

**Figure 4:**
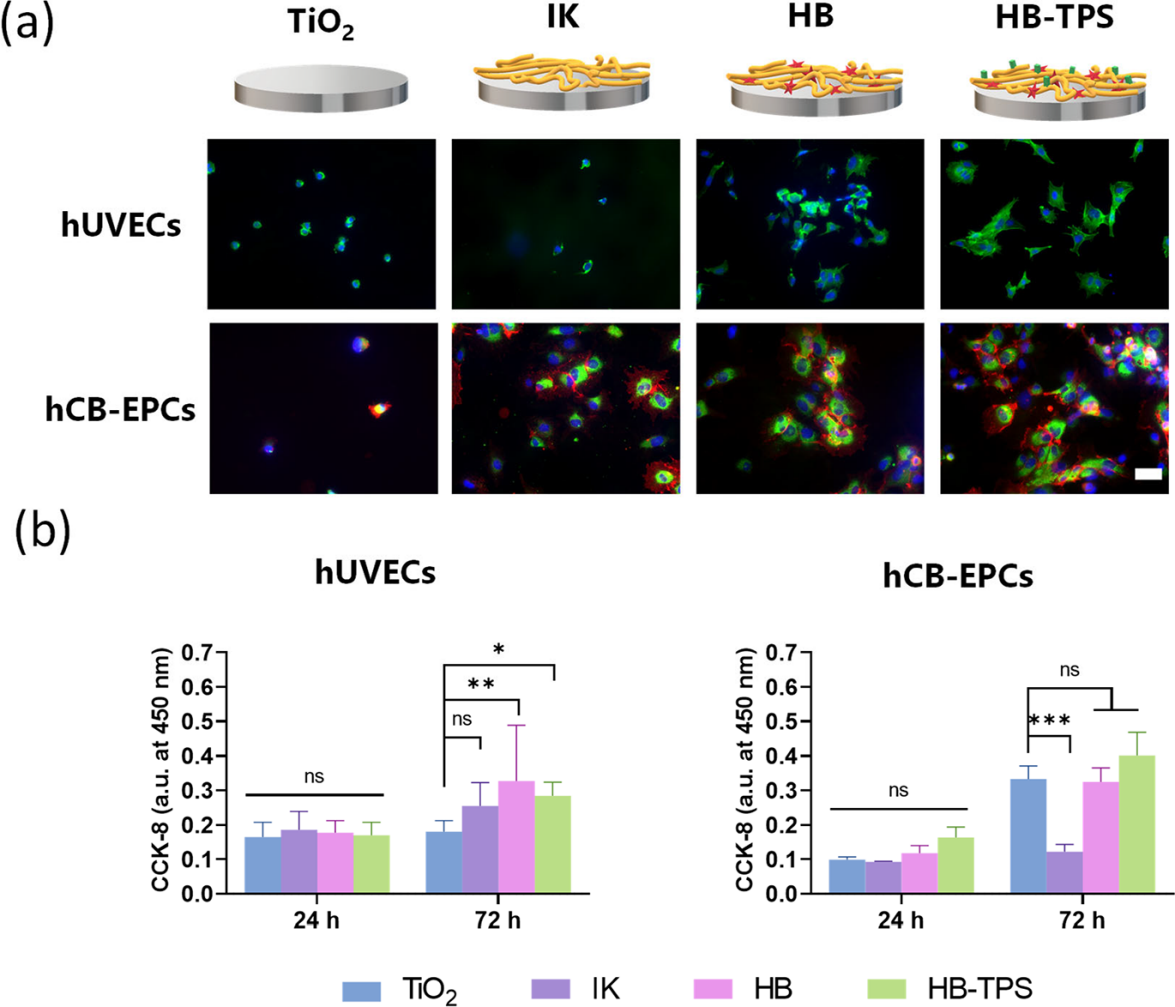
In vitro evaluation of the cellular response to the ELR nanocoatings using primary vascular cell types. (a) Fluorescence images of human umbilical vein endothelial cells (hUVECs), and human cord blood-endothelial progenitor cells (hCB-EPCs) after being incubated for 2 h on TiO_2_ or ELR-coated surfaces. The surfaces were pretreated with a 1% (w/v) bovine serum albumin (BSA) solution to promote specific cell adhesion. hUVECs: blue = nuclei and green = F-actin. hCB-EPCs: blue = nuclei, green = F-actin and red = CD31. Scale bar is 50 μm. (b) Cell proliferation after 24 and 72 h measured by using the CCK-8 assay. Data are represented as mean ± SD, with *n* = 8 for hUVECs (*p < 0.05, **p < 0.01) and *n* = *3* for hCB-EPCs (***p < 0.01).

On pristine TiO_2_ surfaces, cells predominantly exhibited a rounded morphology indicative of poor adhesion. In contrast, cells seeded on the bioactive ELR coatings (HB and HB-TPS) showed an enhanced spreading, with variations depending on the cell line. For hUVECs, both bioactive coatings (HB and HB-TPS) supported better cell adhesion and spreading compared to nonbioactive ELR coating (IK). These findings are consistent with previous studies showing that PRRARV peptide promotes endothelial adhesion by interacting with membrane proteoglycans and indirectly through growth factor binding.^64^ Heparin interacts with fibronectin mainly through this peptide and allows fibronectin to interact with other proteins, including growth factors.^65^ Furthermore, PRRARV can directly interact with cell surface proteoglycans, contributing to cell adhesion.^66,67^ As such, tethering of PRRARV peptide on biomaterial surface can promote the interactions with host cells indirectly through heparin-mediated growth factor binding and directly through proteoglycans.^66^

Interestingly, hCB-EPCs adhered to all ELR-coatings, with a higher number of cells observed on coatings that present the TPS peptide, which promotes the specific binding of EPCs.^51,68^ Surprisingly, even the IK coating, supported significant EPC attachment, albeit at lower levels than HB-TPS (Figure 4a).

To further assess the impact of ELR on endothelialization, proliferation assays were performed (Figure 4b). For hUVECs, ELR coatings containing the heparin-binding peptide PRRARV (HB and HB-TPS) significantly enhanced proliferation compared to pristine TiO_2_, with both coatings showing similar effects. No additive or synergistic effect was observed when both PRRARV and TPS were present in the HB-TPS coating. This indicates that the presence of PRRARV on the coatings was sufficient to promote hUVEC in vitro proliferation.

In contrast, hCB-EPCs exhibited a different trend. After 72 h, the IK coating, which lacks bioactive sequences, exhibited a reduced proliferation compared to TiO_2_. However, the inclusion of peptides such as PRRARV and TPS in the HB-TPS coatings dramatically enhanced cell proliferation, surpassing the levels observed on TiO_2_. Notably, the presence of the TPS peptide on the ELR coatings showed the highest proliferation rates, likely due to the combined functionality of both peptides, which may act additively to support cell adhesion and proliferation de EPCs. These results align with prior studies highlighting the efficacy of the TPS peptide in EPC homing under both in vitro and in vivo conditions.^51,68^

The observed differences in cell proliferation between TiO_2_ and ELR-coated surfaces may be influenced by their distinct surface properties. ELRs are known for their low-fouling characteristics, which reduce nonspecific protein adsorption, thereby minimizing random protein deposition on the surface.^26,60^ In contrast, pristine TiO_2_ surfaces, lacking this low-fouling behavior, may readily adsorb serum proteins, especially under the high serum concentration (20% FCS) used in these assays. This protein adsorption likely creates a provisional ECM that facilitates cell attachment and proliferation, as has been observed in similar systems.^69^ This phenomenon underscores a potential limitation of in vitro assays performed under high serum conditions, but it also highlights the advantages of the bioactive ELR coatings. The incorporation of specific bioactive motifs, such as PRRARV and TPS, compensates for the low-fouling properties of ELRs by actively promoting cell adhesion and proliferation. These motifs not only enhance the functional interaction with endothelial and progenitor cells but also enable controlled cell-surface interactions, which are essential for implant integration.

Given the essential role of healthy endothelium in preventing thrombosis and stenosis, the ability of ELR-based coatings to promote endothelial cell adhesion and proliferation suggests their potential for cardiovascular applications. These results further support the use of ELR coatings for improving the biological performance of cardiovascular implants and other blood-contacting devices, warranting further investigations in ex vivo and in vivo models.

## Conclusions

The study demonstrates the effectiveness of ELR-based coatings for functionalizing biomaterial surfaces through a dual-functional azide-DOPA peptide strategy that uses catechol adhesion chemistry for robust substrate anchoring and bioorthogonal click chemistry. This approach enables to successfully tether ELRs to both metallic (TiO_2_) and polymeric (PET, TPU) surfaces, demonstrating a high versatility in the coating of different devices. The protein-engineered coatings incorporated bioactive peptides, such as PRRARV and TPS, which significantly enhanced endothelial cell and endothelial progenitor cell adhesion and proliferation compared to pristine TiO_2_. Notably, the HB-TPS coating, combining PRRARV and TPS peptides, exhibited the highest efficacy for endothelial progenitor cell proliferation, suggesting that the integration of multiple bioactive sequences creates additive effects for improved endothelialization. The coatings also demonstrated the capacity to bind heparin, indicating their potential to reduce thrombogenicity in blood-contacting applications, but most importantly to interact with other heparin-binding proteins, such as growth factors.

This versatility combined with the ability to present multiple bioactive motifs in a stable and controlled manner, highlights ELR coatings as a powerful platform for improving the biological performance of implants. By mimicking key extracellular matrix properties, these coatings can enhance implant integration and mitigate complications associated with long-term biomedical device use. These findings warrant further exploration in ex vivo and in vivo models to validate their clinical potential in diverse applications, including blood-contacting materials and other permanent implants.

## Supplementary Material



## Notes

Amino acid sequences, molecular weights, and purity data of the ELRs and peptides used, surface bonding performance analysis derived from QCM-D and SPR measurements, MALDI-TOF characterization of ELR modification, AFM-based surface morphology and roughness analysis pre- and postfunctionalization, high-resolution XPS spectra confirming surface functionalization steps, evaluation of ELR coating stability under mechanical and physiological conditions, XPS analysis of other biomaterial substrates functionalized via the same strategy, real-time QCM-D monitoring of multilayer ELR coatings fabricated via layer-by-layer assembly, and in vitro adhesion evaluation of hUVECs and hCB-EPCs on ELR coatings (PDF)
