# Supplementary material for: Bioorthogonal Mussel-Inspired Elastin-like Nanocoatings for Indwelling Devices
Source: ACS Appl Mater Interfaces. 2025 Sep 1;17(36):50279–91. doi: 10.1021/acsami.5c10327 (PMC12442001; doi:10.1021/acsami.5c10327)
Supplement: Supplementary file 1 [file am5c10327_si_001.pdf]

# Supporting Information

## Bioorthogonal mussel-inspired elastin-like nanocoatings for indwelling devices

*Sergio Acosta[1,2]*, Viktoriya Chaskovska[1], Ikram El-Maachi[1], Jenny Englert[3,4], María Puertas-Bartolomé[2], Stefan Jockenhoevel[1], José Carlos Rodríguez-Cabello[2], César Rodriguez-Emmenegger[3,5,6,7], Alicia Fernández-Colino[1]**

1 – Department of Biohybrid & Medical Textiles (BioTex), AME –Institute of Applied Medical Engineering, Helmholtz Institute, RWTH Aachen University, 52074 Aachen, Germany

2 – Bioforge Lab (Group for Advanced Materials and Nanobiotechnology), Laboratory for Disruptive Interdisciplinary Science (LaDIS), CIBER-BBN, Edificio LUCIA, Universidad de Valladolid, Valladolid, Spain

3 – DWI - Leibniz Institute for Interactive Materials, Forckenbeckstraße 50, 52074 Aachen, Germany

4 - Chair of Biotechnology, RWTH Aachen University, 52074 Aachen, Germany

5 - Institute for Bioengineering of Catalonia (IBEC), The Barcelona Institute of Science and Technology (BIST), Carrer de Baldiri Reixac 10-12, 08028 Barcelona, Spain

6 - Institució Catalana de Recerca i Estudis Avançats (ICREA), Passeig Lluís Companys 23, 08010 Barcelona, Spain

7- Biomedical Research Networking, Center in Bioengineering, Biomaterials and Nanomedicine, The Institute of Health Carlos III, 28029 Madrid, Spain

*E-mail: sergio.acosta@uva.es, fernandez@ame.rwth-aachen.de

**Table S1.** Amino acid sequence of the ELRs used in this work.

| Name | Sequence | Theo.[a] $M_W$ (Da) | Exp.[b] $M_W$ (Da) |
|---|---|---|---|
| nHB | MESLLP $([(VPGIG)_2VPGKG(VPGIG)_2)_2$ $VG_5PRRARVG_5$ $[(VPGIG)_2$ VPGKG $(VPGIG)_2])_6V$ | 60403 | 60780 |
| HB | nHB sequence with 9 Lys side chains modified with cyclooctyne groups[b] | 62364 | 62246[c] |
| HB-TPS | Sequence of the HB with 3 TPS peptides anchored to cyclooctyne groups by click chemistry [c] | 66623 | 66623[d] |

[a]The theoretical molecular weight (Theo. $M_W$) was calculated with the ProtParam tool (https://web.expasy.org/protparam/).

[b]The experimental $M_W$ (Exp. $M_W$) was measured by mass spectroscopy (Fig. S1).

[c]An average of 8.3 cyclooctyne groups were incorporated into each ELR chain.

[d]To produce the HB-TPS polypeptide, three TPS peptides (Theo.) were incorporated per molecule, of which an average of 2.46 peptides were introduced.

**Table S2.** Amino acid sequence of the peptides used in this study.

| Name | Sequence | Theo. $M_W$ (Da) | Exp. $M_W$ (Da)[a] | Purity[b] |
|---|---|---|---|---|
| AD | (DOPA)-G-(DOPA)-GGSGGK$(N_3)$-$NH_2$ | 899.23 | 900.12 | 99.59% |

| | | | | |
|---|---|---|---|---|
| TPS | TPSLEQRTVYAKGGGGK($N_3$)-$NH_2$ | 1773.93 | 1774.64 | 92.92% |

[a]Molecular weight ($M_W$) measured by MALDI-ToF. [b]Purity was measured by HPLC by the manufacturer (CASLO ApS, Denmark)

**Table S3.** Bonding performance estimated from QCM-D and SPR results.

| Coating | AD | | HB | | HB-TPS | |
|---|---|---|---|---|---|---|
| | QCM-D | SPR | QCM-D | SPR | QCM-D | SPR |
| [a]Molecules ($\times10^{12}$) / $cm^2$ | 87.7±30.2 | 48.9±12.8 | 7.2±2.9 | 1.7±0.6 | 7.4±4.3 | 1.3±0.8 |
| [b]AD peptides / ELR | - | - | 12.16 | 29.2 | 11.88 | 38.1 |
| [c]Azide / cycooctyne | - | - | 1.46 | 3.52 | 2.04 | 6.52 |

[a] Values represent mean ± SD, expressed as $\times10^{12}$ molecules/$cm^2$. "Molecules" correspond to AD peptides immobilized after the first functionalization step or ELRs after the second step.

[b] "AD peptides / ELR" indicates the estimated number of AD peptides available per immobilized ELR molecule.

[c] "Azide / cyclooctyne" reflects the estimated ratio between azide groups and cyclooctyne groups on the surface calculated from the corresponding surface densities.

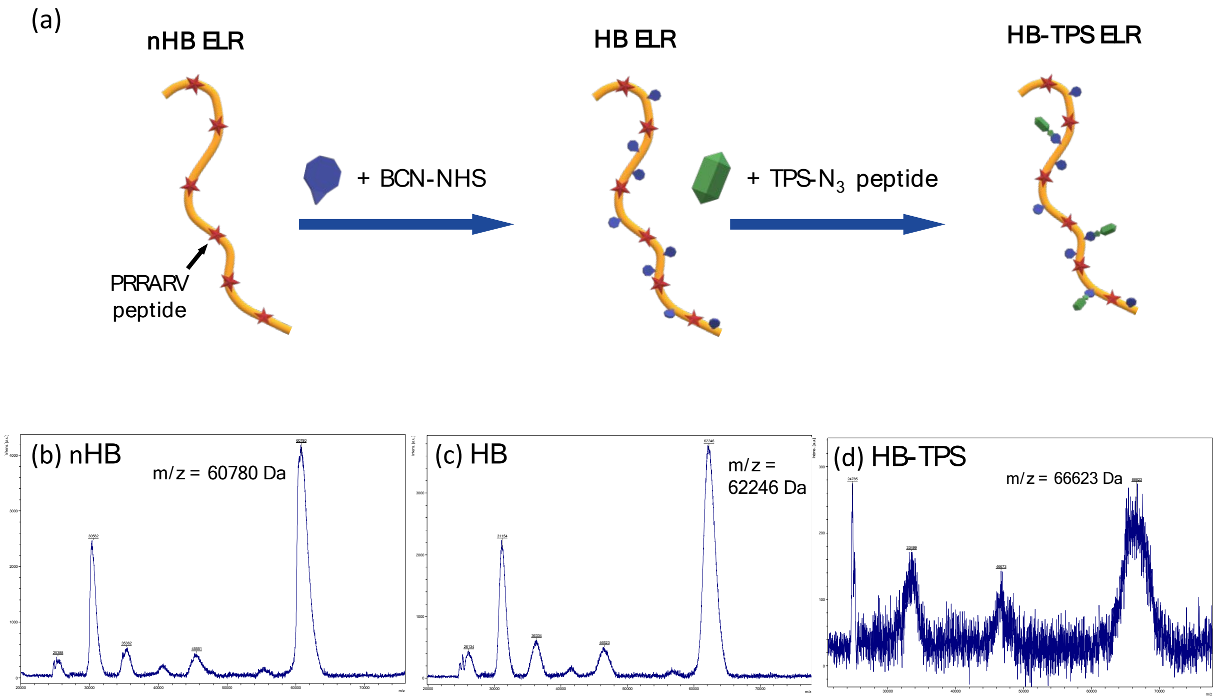


**Figure S1**. (a) Schematic representation of the chemical derivatization of the nHB ELR. The Lys side chains were used to introduce cyclooctyne (BCN-) groups (HB), which in turn served to anchor the cell-adhesive TPS peptide by click chemistry to obtain HB-TPS. The modification of the ELRs was monitored by matrix-assisted laser desorption/ionization time-of-flight (MALDI-TOF): (b) nHB, (c) HB, and (d) HB-TPS.

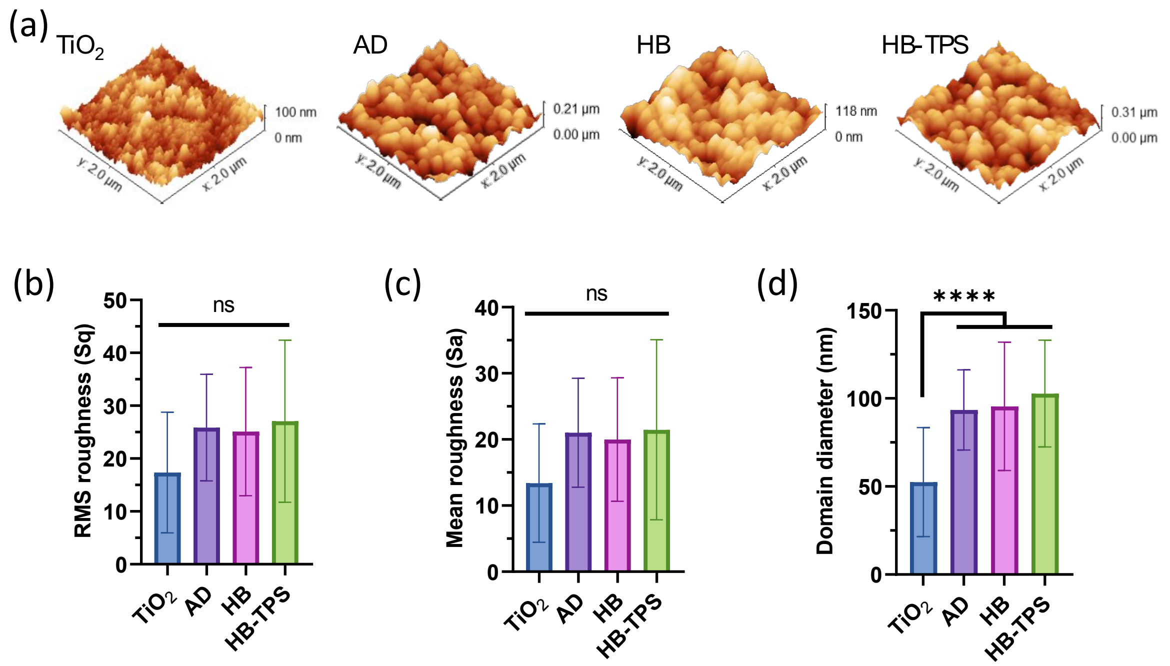


**Figure S2**. Surface morphology characterization of $TiO_2$ substrates before and after functionalization with AD peptide, HB ELR, and HB-TPS ELR coatings. (a) Representative 3D AFM images (2 µm × 2 µm) showing the topography of pristine $TiO_2$ after NaOH etching, AD-modified, HB-coated, and HB-TPS-coated surfaces. Quantitative analysis of surface roughness: (b) RMS roughness (Sq), (c) mean roughness (Sa), and (d) grain diameter for each surface condition (*n = 3*). Error bars represent standard deviation.

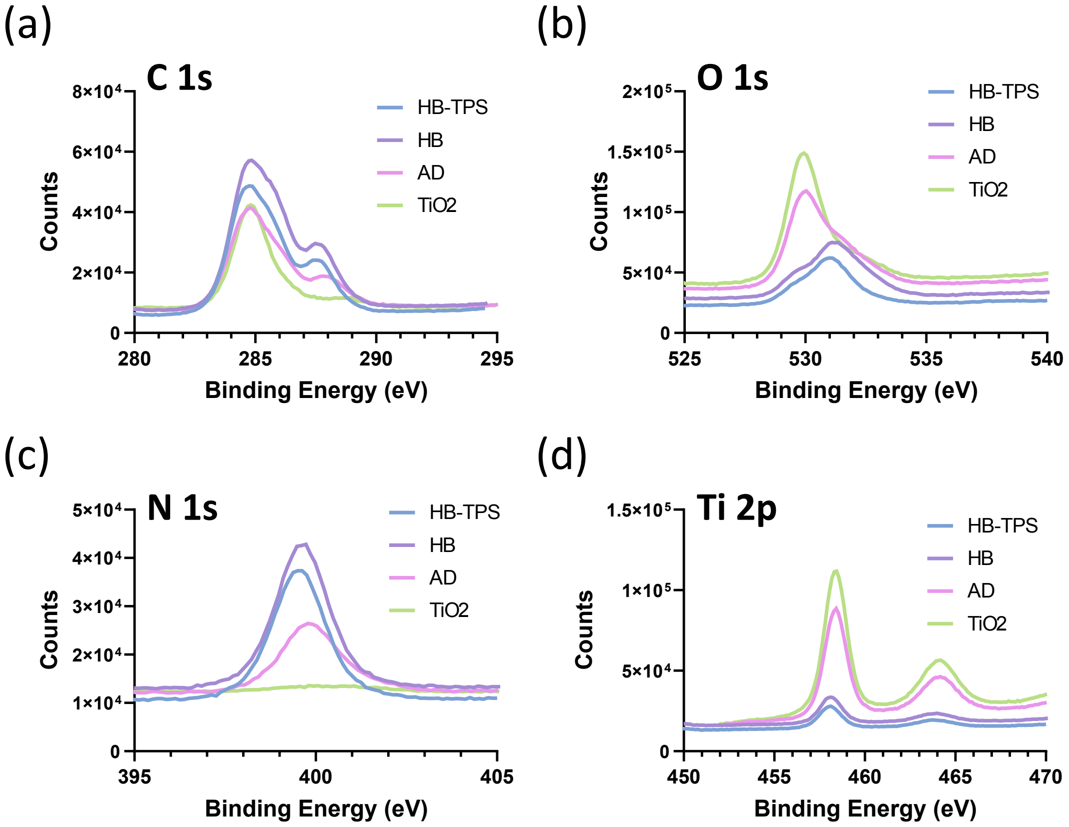


**Figure S3**. High-resolution XPS spectra of the coated surfaces after each functionalization step. (a) C%1s, (b) O%1s, (c) N%1s, and (d) Ti%2p regions for pristine $TiO_2$, AD-modified surfaces, and surfaces coated with HB and HB-TPS ELRs.

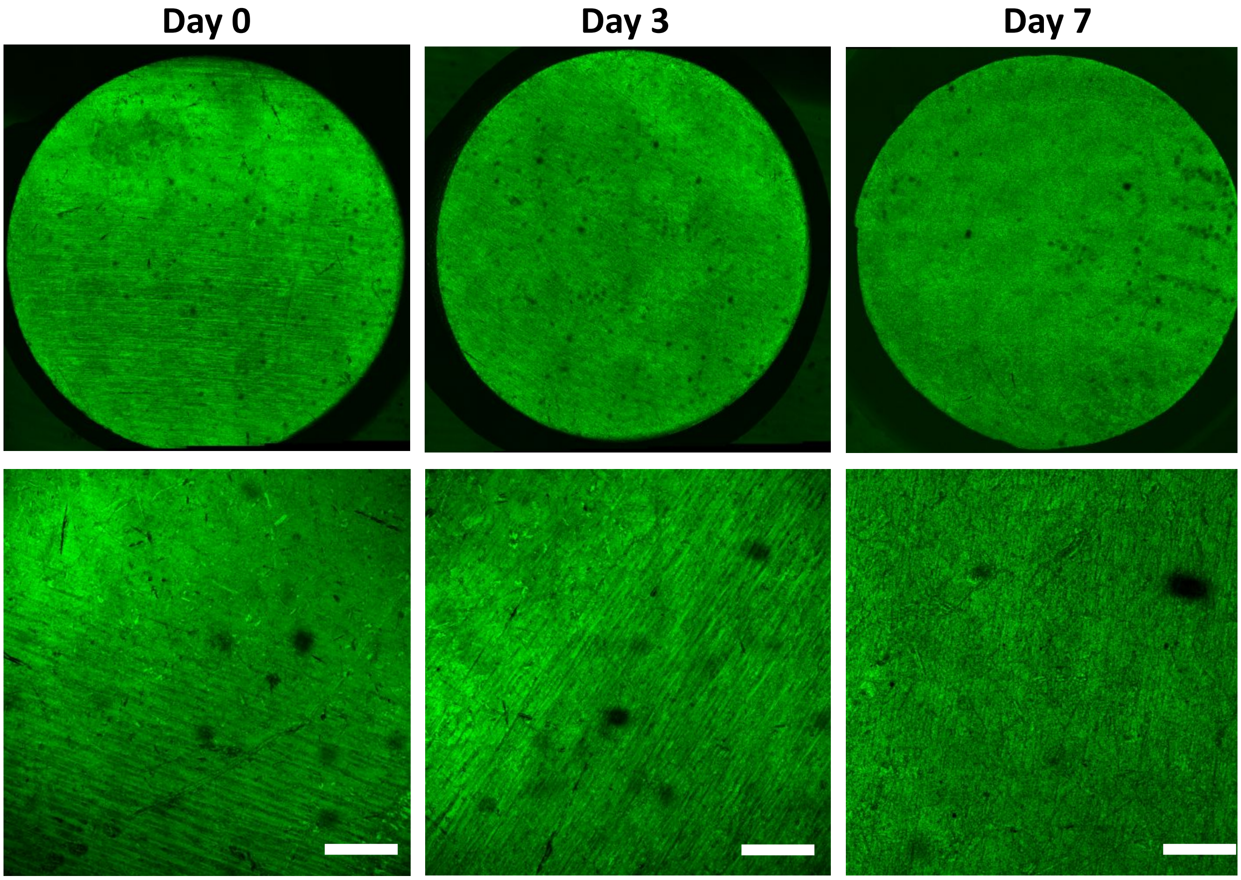


**Figure S4**. Evaluation of coating stability under physiological conditions. FITC-labeled HB ELR nanocoatings were prepared on TiO2 surfaces and subjected to ultrasonication (5 min) followed by incubation in PBS at 37%˚C for 0, 3, and 7 days. Top row: representative confocal images showing the entire coated disc surface. Disc diameter = 6 mm. Bottom row: magnified views highlighting the surface appearance of the fluorescent coating. Scale bar: 200 µm. The uniform fluorescence signal over time confirms the stability and retention of the nanocoating under mechanical and physiological conditions.

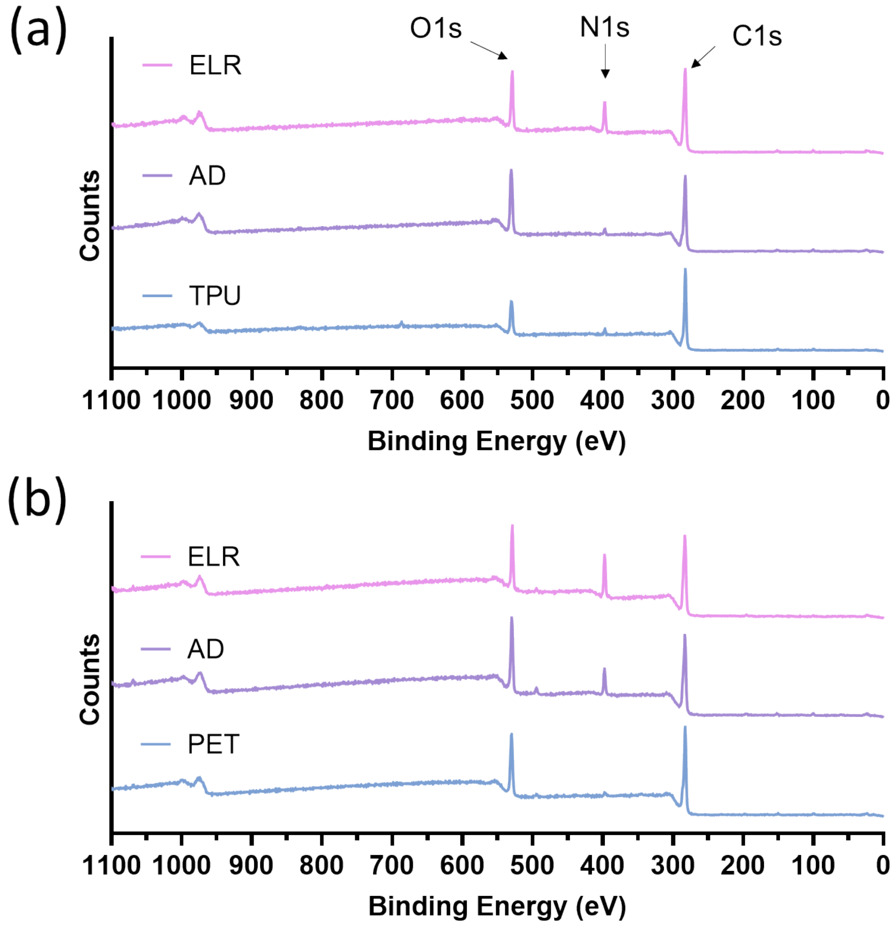


**Figure S5**. XPS spectra of different biomaterials after different modification steps during the fabrication of ELR coatings by using AD peptides: (a) thermoplastic polyurethane (TPU), (b) polyethylene terephthalate (PET).

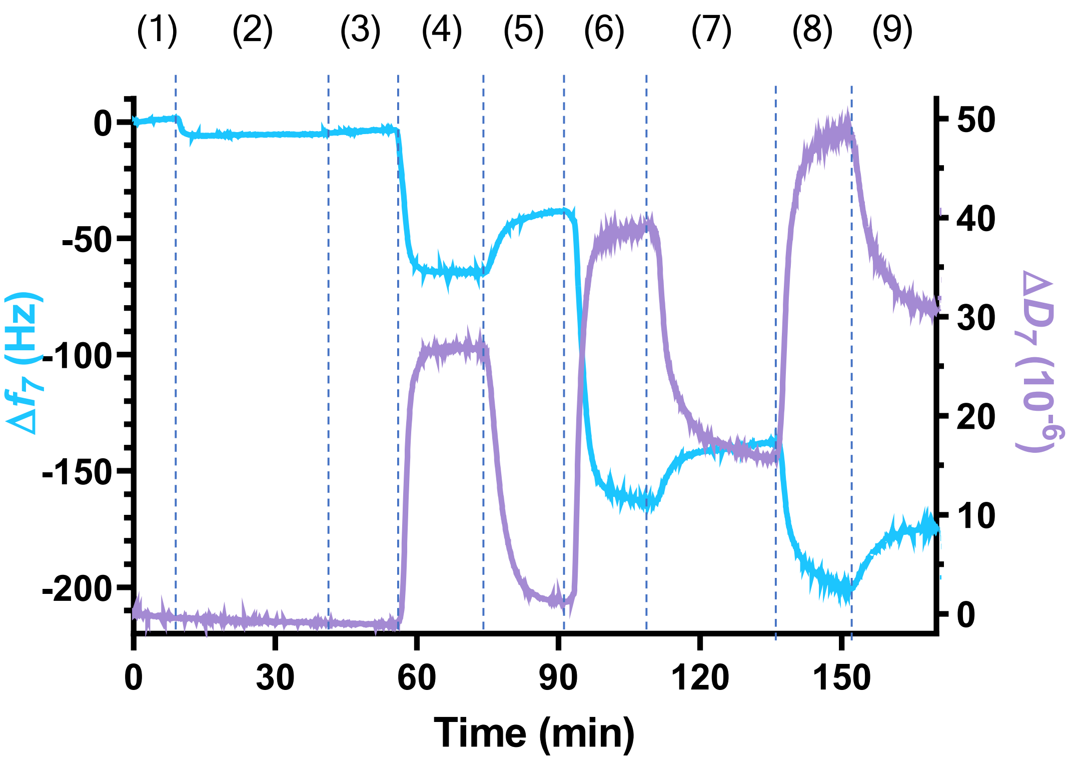


**Figure S6**. Real-time monitoring of the fabrication of multilayered ELR coatings by LbL. The measurement cycle was as follows: (1) baseline recorded in solvent solution (50% ethanol in ultrapure water); (2) addition of the AD peptide to create an azide coating; (i3) rinsing with 50% ethanol; (4) bioorthogonal tethering of the cyclooctyne-bearing HB ELR; (5) rinsing with 50% ethanol; (6) bioorthogonal tethering of the azide-bearing HB ELR; (7) rinsing with 50% ethanol; (8) bioorthogonal tethering of the cyclooctyne-bearing HB ELR; (9) rinsing with 50% ethanol.

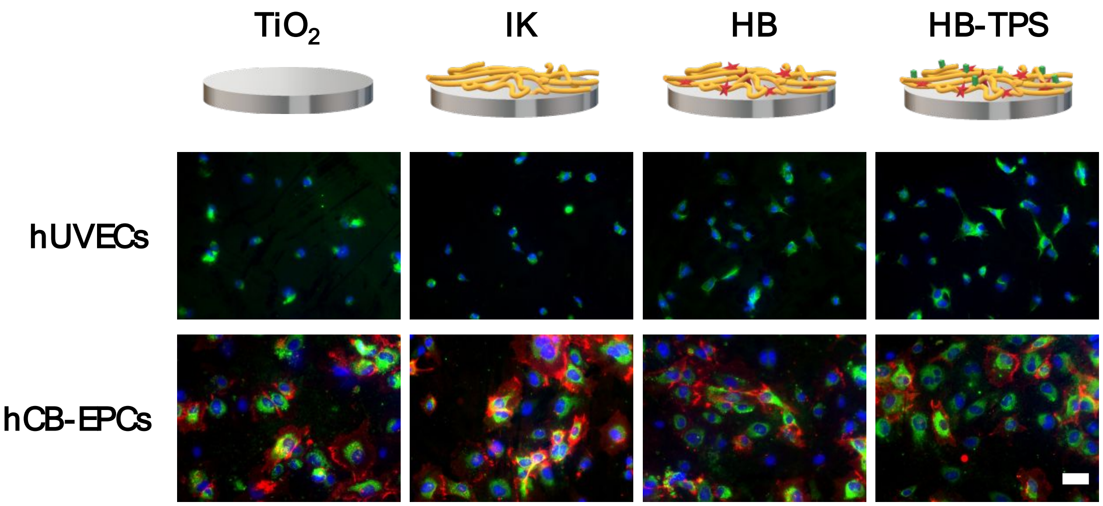


**Figure S7**. *In vitro* evaluation of the adhesion of primary endothelial cell lines on the ELR coatings. Visualization of human umbilical vein endothelial cells (hUVECs), and human cord blood-endothelial progenitor cells (hCB-EPCs) after being incubated for 2 h on $TiO_2$ or ELR-coated surfaces without the pretreatment with 1% (w/v) bovine serum albumin (BSA) solution. hUVECs: Blue= nuclei and Green=F-actin. hCB-EPCs: Blue= nuclei, Green= F-actin and Red=CD31. Scale bar is 50 μm.
